# Transcriptome Analysis Reveals New Insights into the Bacterial Wilt Resistance Mechanism Mediated by Silicon in Tomato

**DOI:** 10.3390/ijms20030761

**Published:** 2019-02-11

**Authors:** Nihao Jiang, Xueying Fan, Weipeng Lin, Guoping Wang, Kunzheng Cai

**Affiliations:** 1 College of Natural Resources and Environment, South China Agricultural University, Guangzhou 510642, China; jnhskip@hotmail.com (N.J.); fanxueying19@163.com (X.F.); linweipeng1986@163.com (W.L.); 2 Institute of Tropical and Subtropical Ecology, South China Agricultural University, Guangzhou 510642, China; 3 Tea Research Institute, Guangdong Academy of Agricultural Science/Guangdong Key Laboratory of Tea Plant Resources Innovation & Utilization, Guangzhou 510640, China; 4 College of Horticulture, South China Agricultural University, Guangzhou 510642, China; gpwang@scau.edu.cn

**Keywords:** silicon, induced resistance, *Ralstonia solanacearum*, tomato, transcriptomics

## Abstract

Bacterial wilt is a devastating disease of tomato caused by soilborne pathogenic bacterium *Ralstonia solanacearum*. Previous studies found that silicon (Si) can increase tomato resistance against *R. solanacearum*, but the exact molecular mechanism remains unclear. RNA sequencing (RNA-Seq) technology was used to investigate the dynamic changes of root transcriptome profiles between Si-treated (+Si) and untreated (−Si) tomato plants at 1, 3, and 7 days post-inoculation with *R. solanacearum*. The contents of salicylic acid (SA), ethylene (ET), and jasmonic acid (JA) and the activity of defense-related enzymes in roots of tomato in different treatments were also determined. The burst of ET production in roots was delayed, and SA and JA contents were altered in Si treatment. The transcriptional response to *R. solanacearum* infection of the +Si plants was quicker than that of the untreated plants. The expression levels of differentially-expressed genes involved in pathogen-associated molecular pattern-triggered immunity (PTI), oxidation resistance, and water-deficit stress tolerance were upregulated in the Si-treated plants. Multiple hormone-related genes were differentially expressed in the Si-treated plants. Si-mediated resistance involves mechanisms other than SA- and JA/ET-mediated stress responses. We propose that Si-mediated tomato resistance to *R. solanacearum* is associated with activated PTI-related responses and enhanced disease resistance and tolerance via several signaling pathways. Such pathways are mediated by multiple hormones (e.g., SA, JA, ET, and auxin), leading to diminished adverse effects (e.g., senescence, water-deficit, salinity and oxidative stress) normally caused by *R. solanacearum* infection. This finding will provide an important basis to further characterize the role of Si in enhancing plant resistance against biotic stress.

## 1. Background

Bacterial wilt caused by *Ralstonia solanacearum* is a destructive disease that limits tomato (*Solanum lycopersicum*) production throughout the world [1]. To date, reliable and effective approaches to control this disease are still lacking [2,3].

Silicon (Si) is the second most abundant element in the Earth’s crust [2,3]. Many studies have documented the beneficial roles of Si in enhancing plant disease (including bacterial wilt) resistance [2,3,4,5,6], but the underlying mechanism remains unclear. The enhanced disease resistance of Si accumulator plants has been linked with mechanical barriers that Si can accumulate and deposit in plants and thereby interfere with the pathogen’s penetration [5,7]. However, this hypothesis cannot adequately explain the role conferred by Si in many Si non-accumulator plants [5,8].

Tomato is a typical Si non-accumulator plant [9]. Several studies showed that Si could enhance the resistance of tomato against bacterial wilt [2,3,9]. Si possibly plays an active role in enhancing tomato resistance to *R. solanacearum* by activating multiple defense responses [2,5]. Ghareeb et al. (2011) reported that the expression of ethylene (ET)- and jasmonic acid (JA)-dependent genes (*JERF3*, *TSRF1*, and *ACCO*) was induced by Si application in *R. solanacearum*-inoculated tomato [9], suggesting that ET and JA pathways participate in Si-induced resistance, but they did not rule out the role of salicylic acid (SA). A recent report has demonstrated that Si induces defense response by increasing the activities of peroxidase (POD), lipoxygenase (LOX), and phenylalanine ammonia-lyase (PAL) in *R. solanacearum*-infected tomato [8]. Our proteomic analysis showed that 26 proteins in the roots were significantly altered by Si in *R. solanacearum*-inoculated tomato plants [3].

Although previous studies have reported the role of Si in enhancing tomato resistance against *R. solanacearum* [3,5,9], the exact molecular mechanism remains unknown. To date, the gene expression changes in Si-treated tomato infected with *R. solanacearum* have been investigated only at a single time point in using microarray analysis, and these studies mainly focused on the tomato stems’ responses to *R. solanacearum* [9,10]. The roots are responsible for perceiving and transmitting signals under various stresses [11]. *R. solanacearum* naturally infects plants through the roots [12], and tomato roots also have an immune system that functions to protect the plant against *R. solanacearum* [13]. Unlike Si accumulator plants (rice, etc.) in which most of Si accumulates above ground, tomato plants contain more Si in roots [2]. Therefore, a transcriptome study of Si-treated tomato roots would be particularly useful to further understand the role of Si in enhancing plant resistance against soilborne disease. 

In this study, we hypothesized that exogenous Si application could enhance tomato plant resistance to *R. solanacearum* via triggering plant immunity response and mediating multiple signaling pathways, and this defense response varies with pathogen infection time. Accordingly, we used RNA sequencing (RNA-Seq) technology to investigate the dynamic changes of transcriptome in Si-treated and non-Si-treated tomato roots at 1, 3, and 7 days post-inoculation (dpi) of *R. solanacearum*. Meanwhile, defense-related enzyme activities and endogenous hormones content (SA, JA, and ET) in roots were also determined to understand how Si influences plant biochemical response and hormones metabolism. 

## 2. Results

### 2.1. Disease Severity and Si Concentration

**Figure 1:**
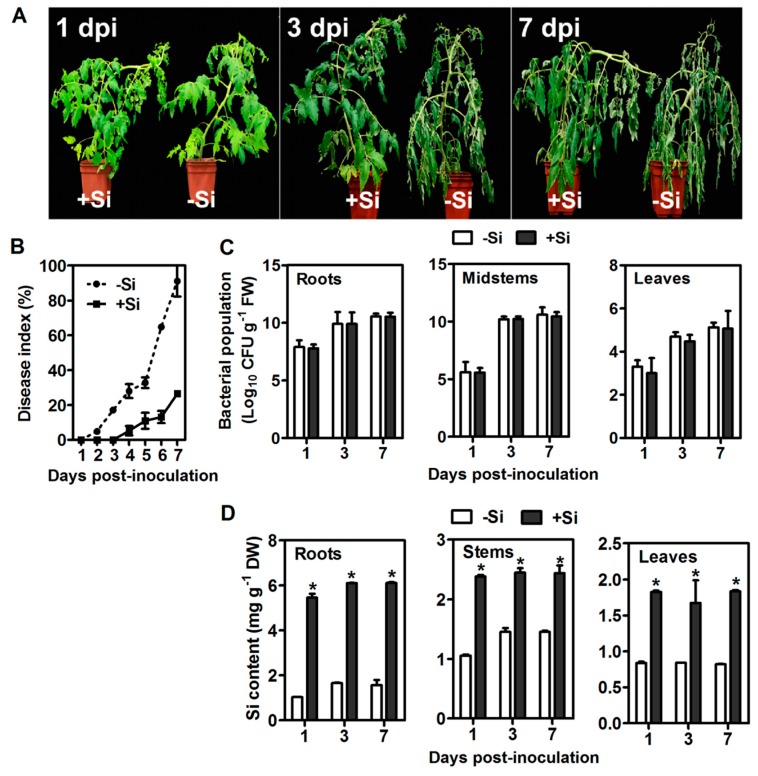
Responses of silicon (Si)-treated (+Si) and non-Si-treated (−Si) tomato plants to *R. solanacearum* infection. (**A**) Disease symptoms. (**B**) Disease index. (**C**) Bacterial population. FW: fresh weight. (**D**) Si content. DW: dry weight. Data presented are means ± standard error (SE) of three replicates. Asterisks denote a significant difference between treatments at the same time-point (Student’s *t*-test, *p* < 0.05).

The wilt symptoms were first observed at 2 dpi in the leaves of no−Si untreated plants, and the plants were completely wilted (80%−100%) at 7 dpi. In the +Si plants, the wilt symptom were delayed and appeared at 4 dpi, and only 20%−30% of the plants were slightly wilted at 7 dpi (Figure 1A). Si pretreatment significantly reduced the disease index by 64.5% at 7 dpi (Figure 1B). No significant difference in the amount of bacterial population was found in tomato plants between treatments (Figure 1C). As a non-Si accumulator, most Si was retained in tomato roots (Figure 1D). 

### 2.2. Biochemical Defense Response

In the +Si treatments, the activities of PAL, polyphenol oxidase (PPO), and POD and the contents of total soluble phenolics (TSPs) and lignin-thioglycolic acid (LTGA) derivatives in roots significantly increased from 1 dpi–3 dpi, peaked at 3 dpi, and decreased thereafter, whereas the activity of LOX increased linearly with inoculation time. The activities of PAL, PPO, and POD and the contents of TSPs and LTGA derivatives were consistently higher in the +Si plants at 2 and 3 dpi than in the –Si plants (Figure S1). The basal resistance response, such as the reinforcement of root cell walls by LTGA derivatives, might partly contribute to tomato resistance against *R. solanacearum*.

### 2.3. Sucrose Metabolism

Jacobs et al. (2012) reported that sucrose is an important nutrient for *R. solanacearum* only during the early stages of root infection of the host plant [12]. Our results showed that sucrose content in leaves increased significantly during 1–2 dpi in the +Si plants in comparison with those in the −Si plants, and no differences were observed at 3 and 7 dpi between treatments. Leaf and xylem sap sucrose concentrations were significantly higher in the +Si2, +Si3, and +Si7 plants than those in the respective –Si plants (Figure S2A). Significantly higher activities of sucrose synthase (SS) and sucrose-phosphate synthase (SPS) in the +Si2, +Si3, and +Si7 plants were also found than in those without Si (Figure S2B,C). Furthermore, Si application continuously increased the activities of SS, neutral invertase (NI), and acid invertases (AI) in leaves after pathogen inoculation, whereas SPS activity decreased (Figure S2).

### 2.4. ET, JA, and SA Contents 

**Figure 2:**
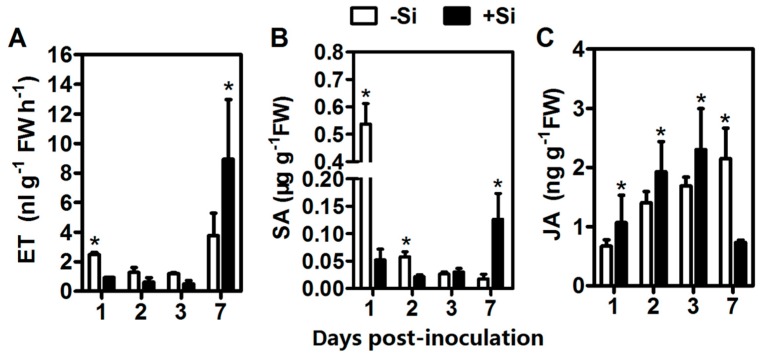
Hormone measurements of silicon (Si)-treated (+Si) and non-Si-treated (−Si) tomato plants after infection. (**A**) ET production. (**B**) SA content. (**C**) JA content. FW: fresh weight. Data presented are means ± standard error (SE) of three replicates. Asterisks denote a significant difference between treatments at the same time-point (Student’s *t*-test, *p* < 0.05).

At 1 dpi, the untreated plants emitted 2.7-fold ET production compared with the +Si plants in response to pathogen infection. However, ET production was 2.3-fold higher in the +Si7 plants than in the untreated plants (Figure 2A). Under the experimental condition, Si treatments delayed the burst of ET production in tomato roots. SA occurred at a significantly higher (tenfold) basal level in the controls at 1 dpi. For the +Si plants, SA content was only significantly higher (7.3-fold) at 7 dpi in comparison with those in the controls (Figure 2B). The JA content in the +Si plants increased gradually from 1 dpi–3 dpi, and it was significantly higher in the +Si1, +Si2, and +Si3 plants compared with the controls; however, JA content in the +Si plants dropped to 0.37-fold of the controls at 7 dpi (Figure 2C). 

### 2.5. RNA-Seq Data Analysis

Approximately 187.21 million reads were generated for the six samples (+Si1, +Si3, +Si7, −Si1, −Si3, and −Si7). The majority of clean reads (more than 89%) were successfully aligned to the tomato reference genome. Approximately 20.35–29.17 million uniquely-mapped reads were retained in the subsequent analysis. An overview of sequencing and mapping results is provided in Table 1. The distribution of gene coverage in each library was analyzed, and the results are presented in Figure S3. Transcriptomic sequences were deposited in the NCBI Sequence Read Archive under Accession Number SRA512164.

### 2.6. Identification and Functional Analysis of Differentially-Expressed Genes 

**Figure 3:**
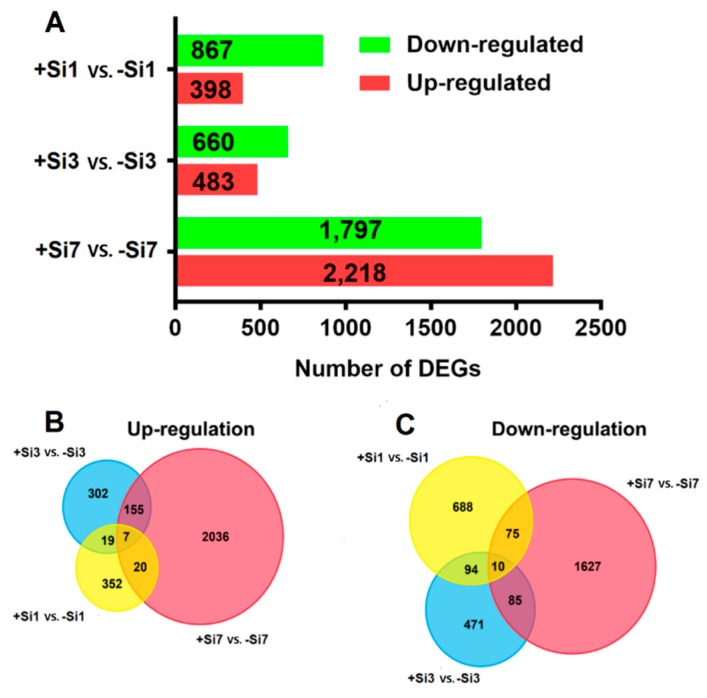
Differentially-expressed genes (DEGs) in different paired-comparisons. (**A**) The numbers of DEGs identified from the three comparisons. (**B**,**C**) Venn diagram for DEGs identified in different comparisons. +Si1, +Si3, and +Si7 represents silicon (Si)-treated (+Si) samples obtained at 1, 3, and 7dpi, respectively; −Si1, −Si3 and −Si7 represents non-Si-treated (−Si) samples obtained at 1, 3, and 7dpi, respectively.

DEGs were identified by three pairwise comparisons of transcriptome datasets (+Si1 vs. −Si1, +Si3 vs. −Si3, and +Si7 vs. −Si7). A total of 1265 DEGs (398 upregulated and 867 downregulated) was identified in the +Si1 vs. −Si1 comparison, 1143 (483 upregulated and 660 downregulated) in the +Si3 vs. –Si3 comparison, and 4015 (2218 upregulated and 1797 downregulated) in the +Si7 vs. −Si7 comparison (Figure 3A). These DEGs are listed in Tables S1–S3, respectively. Scatter plots of DEGs in each comparison are presented in Figure S4. 

PCA analysis showed that the major variation (85.8%) of the transcriptome dataset could be explained by the first two principal components (Figure S5). For the first principal component, the assignment of −Si7 was similar to that of +Si3. The Pearson correlation between −Si7 and +Si3 was higher than that between −Si7 and +Si7 (Figure S6). 

Functional classes of all DEGs were defined using GO terms. The molecular function related to reactive oxygen species (ROS) was significantly enriched among DEGs in the three comparison groups, including “oxidoreductase activity” and “antioxidant activity” (Table S4). KEGG pathway enrichment analysis of all DEGs was then performed. The pathway “phenylpropanoid biosynthesis” was significantly enriched in all comparison groups (Table S5).

Further analysis identified unique and shared DEGs among the three paired-comparisons (Figure 3B,C). Only 17 DEGs were shared among the three groups, indicating significant differences in the transcriptome responses among them. Most of the 17 common DEGs were annotated as predicted proteins (Table S6).

### 2.7. Validation of RNA-Seq Data by qRT-PCR Analysis

**Figure 4:**
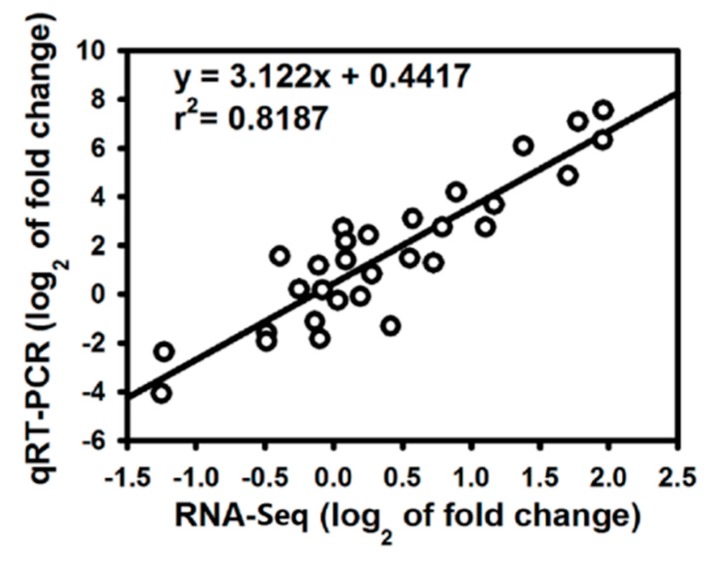
Coefficient analysis of gene expression levels obtained from RNA-Seq and qRT-PCR data. Log_2_ fold change: log_2_ fold-change in gene expression between Si-treated and non-Si-treated samples.

To verify the RNA-Seq results, the expression of 10 genes related to defense response in the +Si and untreated tomato roots at three stages of *R. solanacearum* infection was analyzed by qRT-PCR with three biological replicates. The qRT-PCR data for these genes were significantly correlated with the RNA-Seq results (*r* = 0.905, *p* < 0.001; Figure 4), which indicates that the RNA-Seq results in the present study are reliable. The FPKM values from the RNA-Seq and qRT-PCR results are shown in Figure S7.

### 2.8. Trend Analysis of DEGs

**Figure 5:**
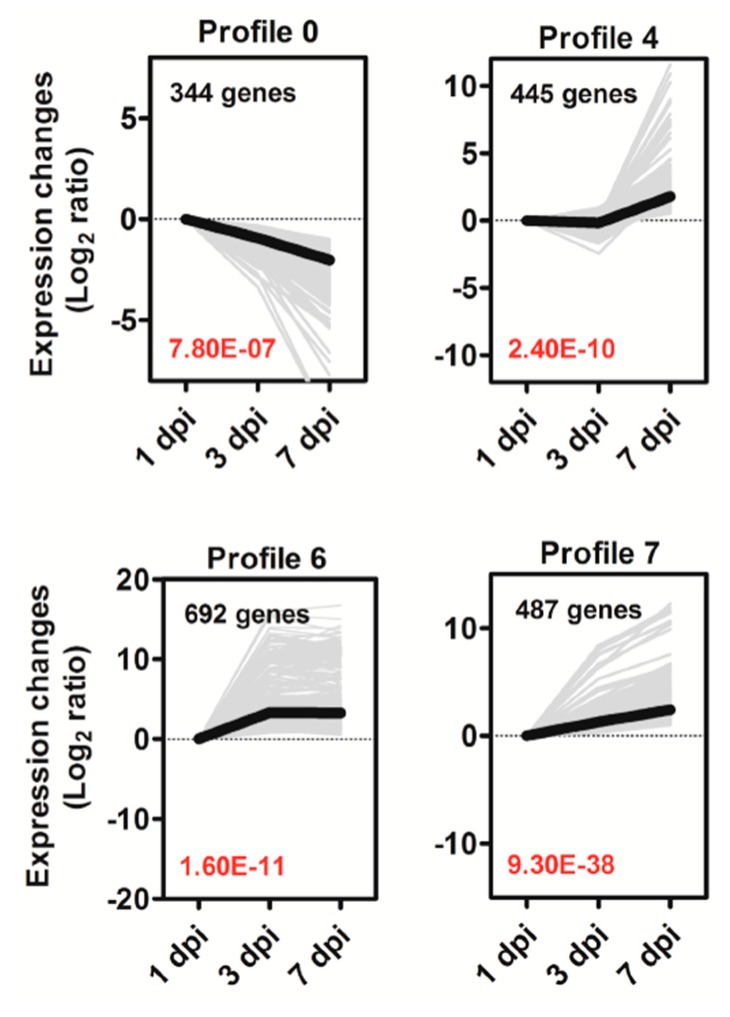
Expression profiles of DEGs in the four main clusters. The top left-hand corner indicates the number of DEGs belonging to the profile. The lower left-hand corner contains the *p*-value of the profile. The gray lines represent the DEGs, and the bold black line represents the expression tendency of all these DEGs. The *x*-axis represents days after *R. solanacearum* inoculation (dpi). The Y-axes represents log_2_ fold change in gene expression between treatments.

To analyze the expression patterns of DEGs playing key roles in Si-mediated tomato resistance against *R. solanacearum*, we performed a trend analysis of DEGs from the three comparison groups (+Si1 vs. −Si1, +Si3 vs. −Si3, and +Si7 vs. −Si7). A total of 3427 DEGs was clustered into eight profiles (Figure 5 and Figure S8, Table S7), but only four significant expression profiles (Profiles 0, 4, 6, and 7, *p* < 0.05) were identified (Figure 5); the changes of these profiles varied with *R. solanacearum* infection time. A total of 344 genes in Profile 0 were continuously downregulated, whereas Profile 7 contained 487 genes that were consistently upregulated. Profile 4 contained 445 genes that were slightly downregulated from 1 dpi–3 dpi and dramatically upregulated from 3 dpi onward. Profile 6 contained 692 genes that were significantly upregulated from 1 dpi–3 dpi, but remained nearly constant from 3 dpi–7 dpi. These different dynamic gene expression patterns further suggest that +Si tomato is resistant to *R. solanacearum* via a highly complex process. 

**Figure 6:**
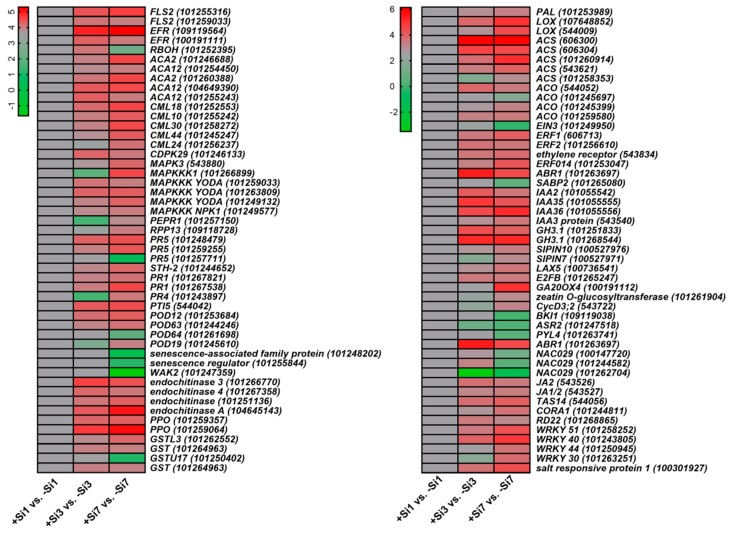
Heat map of DEGs potentially associated with silicon (Si)-mediated tomato resistance against *R. solanacearum*. The columns represent the pairwise comparisons of Si-treated samples (+Si) and their respective non-Si-treated samples (−Si) at three time points post-inoculation. +Si1, +Si3, and +Si7 represent Si-treated samples obtained at 1, 3, and 7dpi, respectively; −Si1, −Si3, and −Si7 represent non-Si-treated samples obtained at 1, 3, and 7dpi, respectively. Relative transcript level was indicated on a color scale from magenta (high) to green (low). DEGs names are on the right side of the figure. Details of the genes shown in heat maps are available in Table S10.

Dynamic processes of +Si tomato plant response to *R. solanacearum* were illuminated by enriched GO terms and KEGG pathways of four significant expression profiles. Several interesting DEGs belonging to Profiles 1, 2, 3, and 5 were manually carried out (Table S7). The enriched GO terms are shown in Table S8. In the KEGG pathway analysis, DEGs involved in phenylpropanoid biosynthesis, plant hormone signal transduction, and plant-pathogen interaction were analyzed in particular (Table S9). On the basis of functional annotation, the candidate DEGs associated with Si-mediated tomato resistance to *R. solanacearum* were identified (Figure 6 and Table S10).

### 2.9. Pattern (PAMP)-Triggered Immunity-Related Genes 

Several PTI-related genes were expressed differentially between the Si-treated and non-Si-treated samples (Figure 6 and Table S10). Pattern recognition receptors (PRRs) are required for the recognition of PAMPs in the early stages of PTI [14]. DEGs encoded flagellin sensing 2 (FLS2) and EF-Tu receptor (EFR) were clustered in Profile 6, whereas FLS2 and EFR are two intensively-studied PRRs [15]. Calcium (Ca^2+^) signaling is an essential second messenger in signal transduction, which plays a vital role in both PTI and effector-triggered immunity (ETI) responses [14]. The mitogen-activated protein kinase (MAPK) module positively regulates the defense [14]. DEGs involved in Ca^2+^ and MAPK signaling pathways, such as *calcium-transporting ATPase 2* (*ACA2*), *ACA12*, *MAPK3*, and *MAPK kinase kinase* (*MAPKKK*), were assigned to profiles that contain genes with upregulated expression at 1 or 3 dpi. Most DEGs identified as WRKY-type transcription factors (TFs) exhibited a similar trend. Ca^2+^ sensor proteins, such as calmodulin-like (CML) (CML44, CML24, etc.), were also induced. The Ca^2+^ signaling mediated via Ca^2+^ sensor proteins may be enhanced by Si treatment. ETI is triggered by R proteins that activate defense reactions upon specific recognition of certain pathogen effectors (Avirulence proteins) [16]. RPP13 (an NBS-LRR type R protein)-like gene was assigned to Profile 4, which is involved in defense responses [17].

### 2.10. Multiple Hormone-Related Genes 

Multiple hormone-related genes were differentially expressed in the +Si plants (Figure 6 and Table S10). In the SA signaling pathway, DEGs encoded pathogenesis-related (PR) genes, such as PR1, PR5, PAL, and STH-2, were continuously upregulated and clustered in Profile 7. PR1, PR2, and PR5 genes are markers of SA-dependent systematic acquired resistance (SAR) and the SA-signaling pathway [18]. PALs, PR1, PR5, and STH-2 (PR protein) also play positive roles in plant disease response [8,19,20]. In Profile 6, one DEG was annotated as transcriptional activator PTI5, which plays a crucial role in *PR* gene regulation [21]. Besides, the DEG encoding salicylic acid-binding protein 2 (SABP2) was continuously downregulated and assigned to Profile 0 (Figure 6 and Table S10).

In ET biosynthesis, most 1-Aminocyclopropane-1-carboxylate synthase (*ACS*) and 1-aminocyclopropane-1-carboxylate oxidase (*ACO*) were upregulated after Si treatment (Figure 6 and Table S10). The ACS and ACO are key enzymes in the biosynthesis of ET [22]. In the ET signaling pathway, ethylene responsive factor 1 (*ERF1*) and ethylene responsive factor 2 (*ERF2*) were upregulated in +Si plants and assigned to Profiles 7 and 6, respectively, whereas ethylene-insensitive 3 (*EIN3*) was downregulated. *EIN3* is necessary for *ERF1* expression, and *ERF1* activation is essential for the ET and JA signaling pathways [23]. *ERF2* serves important functions in the transcriptional regulation of genes related to the JA/ET-mediated defense response pathways [24]. In JA biosynthesis, the DEG encoding LOX was assigned to Profile 7 (Figure 6 and Table S10).

Plant hormones such as auxin/indole-3-acetic acid (IAA), brassinosteroid (BR), cytokinin (CK), gibberellin (GA), and abscisic acid (ABA) play multiple roles in the regulation of plant growth and defense and stress responses [25]. In the auxin signaling pathway, *auxin efflux facilitator PIN10*, *auxin influx carrier LAX5*, and IAA protein were continuously upregulated and assigned to Profile 7, whereas *SlPIN7* was assigned to Profile 4 (Figure 6 and Table S10). Increased expression of *PIN3* and *PIN7* prevents the localized accumulation of auxin and ultimately limits the formation of lateral roots [26]. *LAX3* promotes lateral root emergence in *Arabidopsis* [26]. The DEGs encoding GH3.1 and IAA were assigned to Profile 6 (Figure 6 and Table S10). IAA-amido synthetase GH3 genes are early auxin-responsive genes encoding enzymes that conjugate amino acids to IAA, thereby inhibiting plant growth [27]. Besides, the TF *E2FB*, which mediates auxin distribution [28], was assigned to Profile 6 (Figure 6 and Table S10). 

BRI1 kinase plays an essential role in BR-regulated plant growth and development. Overexpression of the BRI1 gene leads to enhanced root growth and decreased sensitivity to ABA [29]. In the BR signaling pathway, *BRI1 kinase inhibitor 1*, a negative regulator of BRI1 and BR signaling [30], was downregulated and assigned to Profile 0. In GA biosynthesis, the gene *GA20OX4* [31] was upregulated from 3 dpi onward and assigned to Profile 4 (Figure 6 and Table S10).

Zeatin and its derivatives are major components of CK [32]. Zeatin *O-glucosyltransferase* catalyzes the *O*-glucosylation of zeatin to *O*-glucosylzeatin, and *O*-glucosylation of zeatin can either increase or decrease the activity of CK depending on the tissue and stage of development [32]. In CK signaling pathway, one DEG annotated as zeatin *O*-glucosyltransferase-like was upregulated from 3 dpi onward and assigned to Profile 4. DEG encoded CycD3; 2 protein was assigned to Profile 4 (Figure 6 and Table S10), which is involved in mediating the effects of CK [33]. 

ABA stress ripening protein 1 (*ASR1*) expression is induced by ABA, water deficit, and salt-stress [34]. *PYL4* (an ABA receptor) is required for ABA-signaling and ABA-mediated responses, such as stomatal closure [35]. AP2-like ABA repressor 1 (*ABR1*) is a negative regulator of ABA signaling [36]. In the ABA signaling pathway, DEGs encoding ASR2 (homologous to *ASR1*) and PYL4-like were continuously downregulated and assigned to Profile 0, whereas *ABR1* was continuously upregulated and assigned to Profile 7. DEGs annotated as *NAC TF* 29 (*NAC029*) were differentially downregulated, which can induce leaf senescence [37]. Senescence-associated genes (SAGs), such as *senescence-associated family protein* and *senescence regulator*, were also continuously downregulated. The DEG annotated as *JA2* was assigned to Profile 6 (Figure 6 and Table S10). 

### 2.11. Stress-Related Genes

The DEG encoding ABA and environmental stress-inducible protein TAS14 was assigned to Profile 6 (Figure 6), which acts as a reliable biomarker for the level of water-deficit stress in plants [38]. One DEG belonging to Profile 7 was annotated as salt-responsive protein 1 (Figure 6), which is involved in plant adaptation to salt stress [39]. The DEG encoding cold and drought-regulated protein CORA-like was found in Profile 4, which is involved in the control of root adaptation to water and salinity stress [40]. The DEG encoding glutathione S-transferase (GST) U17 (GSTU17)*-*like was continuously downregulated and found in Profile 0, which acts as a negative regulator of salt and drought stress response [41]. In addition, The DEGs encoding dehydration-responsive protein RD22 and Ca^2+^-dependent protein kinase 29 (CDPK29) were assigned to Profile 6 (Figure 6 and Table S10), which is involved in plant adaptation to drought and salinity stress [42,43]. 

The DEGs encoding POD, PPO, and GST were differentially upregulated. One DEG belonging to Profile 7 was annotated as GSTL3, which is associated with tomato resistance to *R. solanacearum* [44]. One DEG with homology to respiratory burst oxidase homolog (*RBOH*) was found in Profile 5 (Figure 6 and Table S10).

## 3. Discussion 

### 3.1. Si Primes Fast Defense Response of Tomato Plants to R. solanacearum Infection

As the second abundant element in soil, the beneficial role of Si in improving plant resistance against biotic and abiotic stresses is widely reported [5]. Evidence showed that Si can induce/prime plant defense and activate signaling pathways [5,7,9]. Our PCA analysis showed that −Si7 and +Si3 were similar for the first principal component (Figure S5), suggesting that the response at the transcriptional level in Si- plants at 7 dpi was similar to that of Si-treated tomato at 3 dpi, indicating that Si treatments induce a quick defense response to *R. solanacearum* infection compared with no Si treatments. Pearson correlation analyses may also suggest this observation (Figure S6). These transcriptional findings are consistent with the results that Si delays the appearance of wilt symptom (Figure 1) and increases the activities of PAL, PPO, and POD and the contents of TSPs and LTGA derivatives (Figure S1).

### 3.2. Si Partially Activated PAMP-Triggered Immune Responses upon R. solanacearum Infection

**Figure 7:**
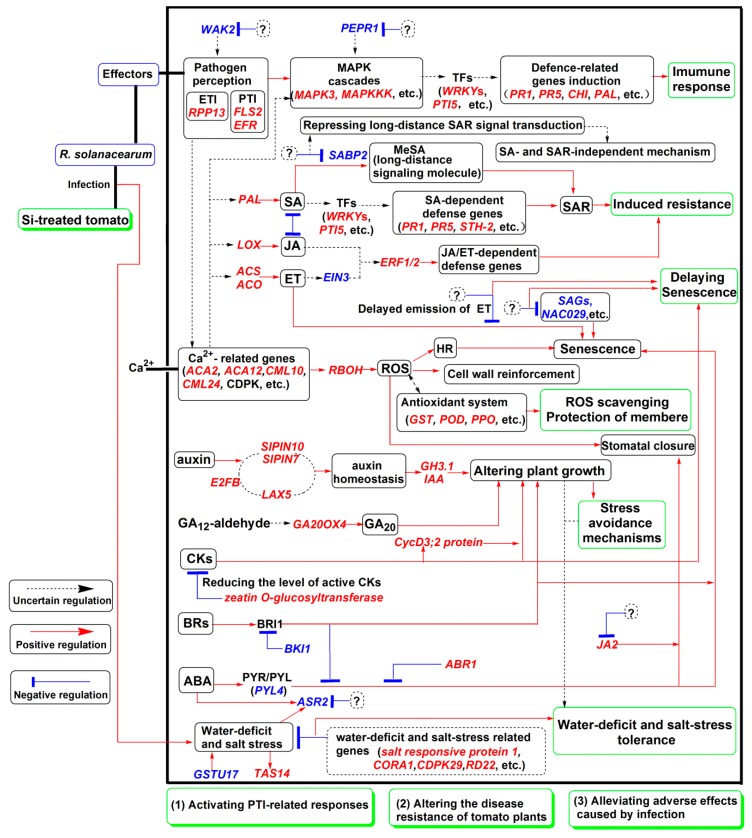
Hypothetical schematic model showing mechanisms involved in silicon (Si)-mediated tomato resistance against *R. solanacearum*. Upregulated genes are marked in red italics, and downregulated genes are marked in blue italics. Black dotted lines with arrows denote uncertain regulation; red lines with arrows denote positive regulation; and blue lines with bars indicate negative regulation. Question marks indicate unclear events. Several crosstalk and feedback mechanisms between multiple pathways are not presented in the model.

Plants have evolved a two-layered immune system that includes PTI and ETI [14,45]. PTI relies on PAMP recognition by PRRs, and the activation of PTI leads to changes in intracellular Ca^2+^ concentration, activation of MAPK cascades, WRKY-type TFs, and transcriptional reprogramming [14,45]. Interestingly, previous studies have reported that the PTI and ETI were partly suppressed in both resistant and susceptible peanut cultivars in response to *R. solanacearum* [19]. In the present study, several PTI-related genes were upregulated in +Si tomato plants (Figure 6 and Table S10). These findings indicated that the activation of PTI-related genes contributes to Si-mediated tomato resistance to *R. solanacearum* (Figure 7). 

### 3.3. Si-Mediated Tomato Resistance to R. solanacearum Involves SA-Dependent and -Independent Mechanisms

Si is known to induce systemic acquired resistance (SAR) upon pathogen infection [7]. SAR is an inducible defense mechanism and is a part of the hypersensitive response (HR) [18,46]. SA is the endogenous signal molecule that is required for the induction of SAR [47]. In the SAR state, plants are primed to speedily and effectively activate defense responses to cope with pathogen attack [18]. In the present study, some SA- and SAR-related genes were upregulated in the +Si plants (Figure 6 and Table S10). These results confirmed that the SA-dependent SAR pathway is involved in Si-mediated tomato resistance to *R. solanacearum* (Figure 7). 

The SAR signal must be transported from infected to uninfected tissue, but SA is not the mobile signal required for the systemic activation of SAR [47]. Methyl salicylate (MeSA) is an essential part of the SAR signal and considered to be a long-distance signaling molecule [48]. SABP2 mediates the hydrolysis of MeSA to SA and is essential for the establishment of SAR [48]. Notably, in +Si tomato, the DEG encoding SABP2 was continuously downregulated (Figure 6 and Table S10). Pathogens manipulate host defense to establish infection successfully [12]. Several possible examples are found in this study. For example, one DEG belonging to Profile 0 was annotated as *wall-associated receptor kinase 2*, which is involved in the early tomato perception of pathogens [11]; *plasma membrane LRR receptor kinase 1* (*PEPR1*), which contributes to the elicitation of PAMP downstream [49], is downregulated (Figure 6 and Table S10). Furthermore, a previous study showed that *R. solanacearum* can suppress the SA defense pathway of *Arabidopsis* [50]. Therefore, these findings indicated that long-distance SAR signal transduction is partly repressed in the +Si plants, but we cannot exclude the possibility that this result is due to pathogen-mediated manipulation of host defense. Besides, SA is also involved in immunity against biotrophic and hemibiotrophic pathogens [51]. *R. solanacearum* is a hemibiotrophic pathogen with biotrophic action during early infection [52]. Our results showed that the disease progression was significantly faster in −Si plants without Si-mediated protection (Figure 1a). Therefore, high basal levels of SA in −Si plants (Figure 2B) may be partly due to the hemibiotrophic nature of *R. solanacearum* [52]. However, in +Si tomato, the SA content was relatively low, from 1 dpi–2 dpi (Figure 2B), and resistance was still retained (Figure 1A). Together, these results may indicate that Si-mediated resistance against *R. solanacearum* in tomato is also involved in an SA- and SAR-independent mechanism (Figure 7).

### 3.4. ET- and JA-Related Pathways Are Involved in Si-Mediated Resistance against R. solanacearum in Tomato

Evidence showed that the JA and ET signaling pathways are involved in the defense against necrotrophic pathogens [53], and both are generally essential for induced systemic resistance [54]. In the present study, most of the ET synthesis-related genes were differentially upregulated (Figure 6 and Table S10), but the release of ET was delayed in Si treatments (Figure 2A). Meanwhile, ET signaling-related genes showed different expression patterns (Figure 6 and Table S10). These results suggest that the ET biosynthesis and signal transduction in +Si tomato are complicated, which may have coordinated and triggered feedback regulation to respond to *R. solanacearum*. In addition, the induction of ET biosynthesis and subsequent activation of the ET pathway were associated with the susceptibility of plants to pathogens and the senescence of tissues [55]. In late stages of infection, hemibiotrophic pathogens may be able to manipulate the plant to produce ET to enter its destructive necrotrophic phase [56] and thus overcome host defenses and cause severe infections. Therefore, these findings also suggest that the delay of ET release in +Si tomato plants is associated with delayed necrotrophic phase progression and symptom appearance (Figure 7). 

LOX catalyzes the key step of lipid peroxidation reaction to produce biologically-active compounds called oxylipins, such as JA, which participates in multiple physiological processes [57]. *LOX* is a JA-mediated defense marker gene [58]. Notably, the DEG encoding LOX was upregulated (Figure 6 and Table S10), which is partially consistent with our result that JA content significantly increased from 1 dpi–3 dpi in +Si tomato (Figure 2C). LOX activity is always associated with lipid peroxidation damage of the cell membrane [8]. In the present study, LOX activity of the +Si plants significantly increased linearly with inoculation time (Figure S1D). Therefore, we cannot conclude that the level of oxidative damage was significantly higher in the +Si plants compared with those in the Si plants. The exact role of LOX in this process might be complicated because its products interact with various metabolites and signaling molecules [57]. Thus, we provided additional evidence to support the concept that the ET- and JA-related pathways are involved to some extent in Si-mediated wilt resistance (Figure 7). 

### 3.5. Si-Mediated Resistance Involves Other Hormone-Mediated Pathways

Auxin influx and efflux carriers regulate auxin distribution [59]. In our study, auxin-homeostasis-related DEGs were assigned into different expression patterns, *PIN10*, *LAX5,* and IAA protein were continuously upregulated after Si treatment (Figure 6 and Table S10). Notably, previous studies have shown that Si treatments could increase auxin accumulation and improve root traits upon pathogen infection [60,61], and morphological improvement of roots was beneficial to stress avoidance [62]. Furthermore, alteration of auxin pathways was also associated with resistance of tomato to *R. solanacearum* [13]. Combined with the above results, auxin homeostasis is regulated in a complex manner in Si treatment, and this process may confer an improved adaptation to multiple stresses, which may positively contribute to Si-mediated tomato resistance against *R. solanacearum* (Figure 7).

ABA is a general inducer of senescence, and the onset of senescence is associated with stomatal closure [63]. In the present study, ABA signaling and SAGs were suppressed to some extent in Si treatment (Figure 7). *JA2* is essential for ABA-mediated stomatal closure [64]. Our results found that *JA2* was not consistently upregulated from 3 dpi–7 dpi, and the expression level of *JA2* was suppressed to some extent (Figure 6 and Table S10). Interestingly, previous studies have shown that net photosynthetic rate and stomatal conductance are significantly high for +Si and *R. solanacearum*-inoculated tomato plants at 7 dpi [60,61]. Thus, suppression of ABA signaling and ABA-mediated stomatal closure in +Si tomato may contribute to tomato plant resistance (Figure 7).

CKs can increase stomatal aperture and/or delay ABA-induced stomatal closure and senescence [65], while BRs exert the role of promoting senescence [66]. In the present case, BR signaling pathways and CK-related pathways are indirectly enhanced and activated, respectively (Figure 7). The roles of BR and CK in this process are complicated by their diverse functions.

Taken together, our results suggest that Si-mediated resistance against *R. solanacearum* in tomato involves multiple hormonal pathways (Figure 7).

### 3.6. Si Treatment Alleviates Water Deficit, Salt, and Oxidative Stresses Caused by Infection

Si can enhance water stress tolerance, as well as improve salinity tolerance in plants [67], while pathogen infection frequently accompanies abiotic stresses such as water and salt stresses [68]. In the present study, DEGs associated with tolerance and adaptation to water-deficit and salinity stress were upregulated upon *R. solanacearum* inoculation (Figure 6 and Table S10). Sucrose content was almost maintained at a significantly higher level for +Si plants compared with the controls (Figure S2). The activities of sucrose metabolism-related enzymes including SS, NI, and AI in leaves were increased in the +Si plants, whereas that of SPS decreased (Figure S2B–E). Similar results were obtained in the tomato plant response to water and salt stresses, and the authors hypothesized that sucrose and these enzymes contribute to alleviating water stress [69]. Gómez-Ariza et al. (2007) found that a high level of endogenous sucrose can be responsible for the early and strong expression of rice defense genes [70]. Combined with these findings, high sucrose contents and activities of sucrose metabolism-related enzymes may contribute to alleviating the water-deficit stress caused by infection, thereby indirectly and partially increasing the tolerance of tomato to bacterial wilt. 

The rapid ROS burst was considered as an early plant response to pathogen infection [54,55]. GST and POD play crucial roles in scavenging ROS, thereby alleviating cell membrane oxidative stress [71]. PPO is an antioxidant defense enzyme and can affect local levels of ROS [71]. In the present study, *POD*, *PPO*, and *GST* genes were differentially upregulated (Figure 6 and Table S10). PPO and POD activities were also increased in +Si tomato (Figure S1). Thus, mitigation of oxidative stress and stabilization of membranes by various activated antioxidant enzymatic systems might partly contribute to bacterial wilt resistance. In addition, ROS act as secondary messengers that regulate various processes in plants from development to stress response [71]. RBOH is involved in ROS production during plant response to stresses [72]. In the present study, one DEG with homology to *RBOH* was induced (Figure 6 and Table S10), suggesting that ROS may be an active signal in Si-mediated tomato resistance (Figure 7). This result also suggests that the balance between the ROS and antioxidant system is complex. 

## 4. Conclusion

Transcriptional response to *R. solanacearum* infection in Si-treated tomato was faster. Si treatment affects the regulation of many DEGs involved in phytohormone synthesis, hormone homeostasis, signal transduction, pathogen resistance, stress adaptation and tolerance, oxidation resistance, and senescence regulation. Si-mediated resistance involves mechanisms other than SA- and JA/ET-mediated stress responses. Si treatment may enhance tomato resistance to *R. solanacearum* infection in three ways: activating PTI- related responses; altering disease resistance and tolerance of tomato plants by influencing multiple hormone (e.g., SA, JA, ET, and auxin) signaling pathways; and alleviating adverse effects (e.g., senescence, water-deficit, and oxidative stress) caused by infection. The proposed hypothetical model may advance our understanding of the role of Si in priming plant resistance to pathogens at molecular level. 

## 5. Materials and Methods 

### 5.1. Plant Materials and Treatments 

The bacterial wilt-susceptible tomato genotype HYT was used in this study. HYT seeds were provided by Professor Wang (College of Horticulture, South China Agricultural University, China), and were originally collected in Guangzhou, China. Seeds were germinated and grown as described by Chen et al. (2015) [3]. Healthy seedlings at the three-leaf stage were transplanted to a pot (170 mm diameter × 165 mm height) filled with a peat-based substrate (Klasmann, Lithuanian Peat Moss, Germany). The plants were maintained in a growth chamber with growth conditions as described by Chen et al. (2015) [3]. The plants were subjected to two treatments: (i) −Si treatment: plants watered with nutrient solution without soluble Si and inoculated with *R. solanacearum* (as controls); (ii) +Si treatment: plants watered with nutrient solution containing 2 mM potassium silicate and inoculated with *R. solanacearum*. Three replicates were established per treatment, and each replicate consisted of 12 plants. For −Si treatment, potassium chloride was used to replenish potassium. The plants were irrigated daily with 30 mL of the corresponding solution (pH 6.5).

### 5.2. Inoculation of R. solanacearum

A highly virulent necrotrophic *R. solanacearum* strain that belongs to race 1 biovar 3 was used for all inoculations. Bacteria were grown on TTC medium for 2 days at 30 °C. Tomato plants at the six-leaf stage were inoculated, and 15 mL of the pathogen suspension (10^8^ CFU mL^−1^) were infused into each pot. Plant samples were collected at 1, 2, 3, and 7 dpi, and three randomly-selected individuals per time point and per type of treatment were used for further analysis. For the +Si treatment, the samples from 1, 2, 3, and 7 dpi were denoted as +Si1, +Si2, +Si3, and +Si7, respectively; for the −Si treatment, they were denoted as −Si1, −Si2, −Si3, and −Si7, respectively.

### 5.3. Bacteria Quantification, Symptom Evaluation, and Si Content Measurement of Plants

Leaves, stems, and roots of randomly-selected plants were sampled at 1, 3, and 7 dpi for quantification of *R. solanacearum*. Bacteria quantification was performed as described by Dannon et al. (2004) [2]. Disease severity and disease index were assessed daily after inoculation with *R. solanacearum* as described by Chen et al. (2015) [3]. The concentration of Si in tomato leaves, stems, and roots were determined at 1, 3, and 7 dpi in accordance with the methods described by Dannon et al. (2004) [2].

### 5.4. RNA Sequencing Library Construction and Illumina Sequencing

Total RNA from Si-treated root samples (+Si1, +Si3, +Si7) and no−Si treated samples (−Si1, −Si3, −Si7) was extracted separately using Column Plant RNAOUT (Tiandz, Beijing) in accordance with the manufacturer’s instructions. For each sample, equal amounts of RNA (20 μg) extracted from the three plants were pooled for cDNA library construction and qRT-PCR analysis. The construction of cDNA libraries and RNA-Seq was performed by Genedenovo Bio-Tech Co., Ltd. (Guangzhou, China). The library was sequenced on an Illumina HiSeq™ 4000 platform with paired-end sequencing reads (2 × 100 bp). 

### 5.5. Bioinformatic Analyses of Transcriptome Data

Raw sequences with adaptors and unknown nucleotides above 5% or those that were of low quality were removed to obtain clean reads. The clean reads were mapped to the tomato reference genome [73] using Tophat2, with Bowtie2 [74]. These mapped reads were used for transcript assembly and abundance estimation by Cufflinks v2.2.1 [75]. The raw gene expression data were normalized using fragments per kilobase of exon per million mapped fragments (FPKM). Genes were considered significantly differentially expressed if the absolute log_2_-fold change >1 and the false discovery rate < 0.05. The expression patterns of DEGs were analyzed using Short Time-series Expression Miner (STEM) [76]. The clustered profiles with *p*-value <0.05 were considered significantly expressed. GO terms and KEGG pathways with corrected *p*-value ≤0.05 were considered significantly enriched. Pearson correlation coefficient analysis and PCA on all samples were performed by R package (Version 2.15.3).

### 5.6. Measurements of Hormone Contents

The ET, SA, and JA contents in fresh root tissues of each treatment at 1, 2, 3, and 7 dpi were measured. For determination of ET, 0.5 g of roots were transferred to 2 mL glass vials, and all bottles were sealed for 8 h. Then, 1 mL of gas was obtained by an air-tight syringe to analyze ET content by an Agilent 6890N gas chromatograph system with a flame ionization detector using an HP5 column (30 m × 0.25 mm). The operating conditions of the GC were as follows: injection volume, 1 mL; initial column temperature, 50 °C for 3 min; temperature increase rate, 15 °C·min^−1^; final column temperature, 250 °C for 5 min; carrier gas (nitrogen) flow rate, 40 mL min^−1^; hydrogen flow rate, 40 mL·min^−1^; air flow rate, 400 mL min^−1^; temperature of injection and detection ports, 250 °C. 

SA and JA contents were determined via ultra-performance liquid chromatography-tandem mass spectrometry. For extraction of SA and JA, root tissues (0.5 g) were ground, suspended in 1 mL of extraction solvent (89.9% MeOH: 9.9% H_2_O: 1% acetic acid), and then stored at 4 °C for 24 h. The supernatants were filtered using nylon syringe filters (0.22 μm), and a Waters ACQUITY UPLC system (Waters, Milford, MA, USA) was used with a Waters ACQUITY UPLC BEH C_18_ column (2.1 × 100 mm, 1.7 μm). The mobile phases consisting of Mobile Phase A (water with 1% formic acid) and Mobile Phase B (methanol with 1% formic acid) were used with a gradient elution of A/B (v/v) from 60:40 (0–0.5 min, hold for 0.5 min), 60:40–40:60 (0.5–4 min), 40:60–60:40 (4 to 4.5 min), and 60:40 (4.5–6 min, hold for 1.5 min) at a flow rate of 0.40 mL/min. The column temperature was 35 °C, and the injection volume was 10 μL. The UPLC system was coupled to Waters Quattro Premier XE in the multiple reaction monitoring mode. The electrospray ionization (ESI) source was operated in both positive and negative ion modes. During the first time segment (0–4 min), SA was analyzed in the positive ESI mode. Within the second time segment (4.2–5.5 min), JA was analyzed in the negative ESI mode. The operational parameters of ESI source were as follows: capillary potential = 3000 V, source temperature = 150 °C, desolvation temperature = 450 °C, cone gas flow = 50 L·h^−1^, desolvation gas flow = 600 L·h^−1^, and drying gas flow = 15 L·min^−1^. The precursor ions, product ions, and MS/MS parameters are displayed in Table S11. All experiments were repeated at least three times with similar results.

### 5.7. Enzyme Activity

The activities of POD, PPO, PAL, and LOX in the roots were determined at 1, 2, 3, and 7 dpi using methods described previously [8,77]. The activities of SS, SPS, AI, and NI in the leaves at each time point were determined in accordance with the methods described by Lu et al. [69]. Experiments were performed at least three times.

### 5.8. Determination of Total Soluble Phenolics, Lignin, Lignin-Like Phenolic Polymers, and Sucrose

The amounts of TSPs and lignin in roots of each treatment at 1, 2, 3, and 7 dpi were determined following previously-described methods [78]. Lignin was quantified by measuring the amounts of LTGA derivatives. The sucrose concentrations in the roots, leaves, and xylem sap of each treatment at 1, 2, 3, and 7 dpi were quantified by using a sucrose assay kit (Nanjing Jiancheng Bioengineering Institute, Nanjing, China) following the manufacturer’s recommendations. Xylem sap was collected as described by Jacobs et al. (2012) [12]. Each experiment was repeated at least three times with similar results.

### 5.9. Validations of RNA-Seq Data by qRT-PCR

qRT-PCR was performed to validate the RNA-Seq results for 10 gene transcripts. The purified RNA (1 μg) was reverse transcribed to cDNA using the FastQuant RT kit (with gDNase) (TIANGEN, Beijing, China). All qRT-PCR reactions were performed in a 20-μL volume composed of 4 μL of cDNA, 0.6 μL of each primer (10 µM µL^−1^), 4.8 μL of sterile water, and 10 μL of qPCR master mix in the ABI Step One Plus Real-Time PCR System (Applied Biosystems, Foster City, CA, USA). The amplification cycling program was as follows: 90 s at 95 °C, followed by 40 cycles of 95 °C for 5 s, 60 °C for 15 s, and 72 °C for 20 s. All primers for qRT-PCR are listed in Table S12. The relative expression levels of the genes were normalized to phosphoglycerate kinase [79] and calculated using the 2^-△△Ct^ method [80]. qRT-PCR analysis was conducted with three technical replicates.

## Figures and Tables

**Table 1:** Summary of RNA-Seq and mapping results.

Samples	Raw Reads	Clean Reads	Q30 (%)	Number of Mapped Reads	Number of Uniquely-Mapped Reads
+Si1	29,666,186	29,302,142	92.72%	26,617,653	26,345,883
+Si3	32,098,976	31,368,678	92.11%	28,055,639	27,773,995
+Si7	29,996,152	23,124,144	92.39%	20,527,662	20,353,204
−Si1	31,207,574	30,772,320	92.23%	27,712,701	27,451,003
−Si3	29,908,868	29,314,980	92.23%	26,107,503	25,872,901
−Si7	34,328,542	33,053,040	92.04%	29,446,516	29,168,022

Note: The Q30 percentage is the proportion of nucleotides with a quality value >30. +Si1, +Si3 and +Si7 represent silicon (Si)-treated (+Si) samples obtained at 1, 3 and 7 dpi, respectively. −Si1, −Si3 and −Si7 represent non-Si-treated (−Si) samples obtained at 1, 3 and 7 dpi, respectively.

## Supplementary Materials

Supplementary materials can be found at http://www.mdpi.com/1422-0067/20/3/761/s1. Table S1. List of the different expressed genes in the +Si1 vs. −Si1 group. Table S2. List of the different expressed genes in the +Si3 vs. −Si3 group. Table S3. List of the different expressed genes in the +Si7 vs. −Si7 group. Table S4. Significantly-enriched GO terms of DEGs. Table S5. Significantly-enriched KEGG pathways of DEGs. Table S6. List of common differentially-expressed genes in the paired-comparison groups. Table S7. The DEGs in each profile. Table S8. Gene ontology (GO) enrichment analysis of four significant clusters. Table S9. KEGG enrichment analysis result of selected pathways in four significant clusters. Table S10. DEGs potentially associated with Si-mediated tomato resistance to *R. solanacearum*. Table S11. MS/MS parameters for determination of phytohormones. Table S12. List of primers used for qRT-PCR experiments. Figure S1. Effect of Si treatment on resistance-related enzymes, total soluble phenols (TSPs), and lignin-thioglycolic acid (LTGA) derivatives in tomato root. Figure S2. Effect of Si treatment on sucrose concentration and activities of enzymes related to sucrose metabolism in tomato at different time points after infection. Figure S3. The distribution of gene coverage in six libraries. Figure S4. Scatter plot of differentially-expressed genes in each comparison. Figure S5. Principal component analysis (PCA) for all RNA-Seq samples. Figure S6. The Pearson correlation coefficients between six samples. Figure S7. Expression of the selected 10 genes revealed by RNA-Seq and qRT-PCR. Figure S8. Expression profiles of the four statistically non-significant clusters (Profile 1, 2, 3, and 5).

## Abbreviations

SA	Salicylic acid
ET	Ethylene
JA	Jasmonic acid
PTI	Pathogen-associated molecular pattern-triggered immunity
POD	Peroxidase
ETI	Effector-triggered immunity
LOX	Lipoxygenase
PAL	Phenylalanine ammonia-lyase
PPO	Polyphenol oxidase
TSPs	Total soluble phenolics
LTGA	Lignin-thioglycolic acid
SS	Sucrose synthase
SPS	Sucrose-phosphate synthase
AI	Acid invertases
NI	Neutral invertase
SAR	Systematic acquired resistance
MAPK	Mitogen-activated protein kinase
CML	Calmodulin-like
ACS	1-Aminocyclopropane-1-carboxylate synthase
ACO	1-Aminocyclopropane-1-carboxylate oxidase
ERF	Ethylene responsive factor
IAA	Indole-3-acetic acid
BR	Brassinosteroid
CK	Cytokinin
GA	Gibberellin
ABA	Abscisic acid
SAGs	Senescence-associated genes
GST	Glutathione S-transferase
ROS	Reactive oxygen species
