# Supplementary material for: Transcriptome Analysis Reveals New Insights into the Bacterial Wilt Resistance Mechanism Mediated by Silicon in Tomato
Source: Int J Mol Sci. 2019 Feb 11;20(3):761. doi: 10.3390/ijms20030761 (PMC6387441; doi:10.3390/ijms20030761)
Supplement: Supplementary file 1 [file ijms-20-00761-s001.zip › Supplementary Figures S1-S8.pdf]

Transcriptome analysis reveals new insights into bacterial wilt resistance mechanism mediated by silicon in tomato

Nihao Jiang , Xueying Fan, Weipeng Lin , Guoping Wang and Kunzheng Cai

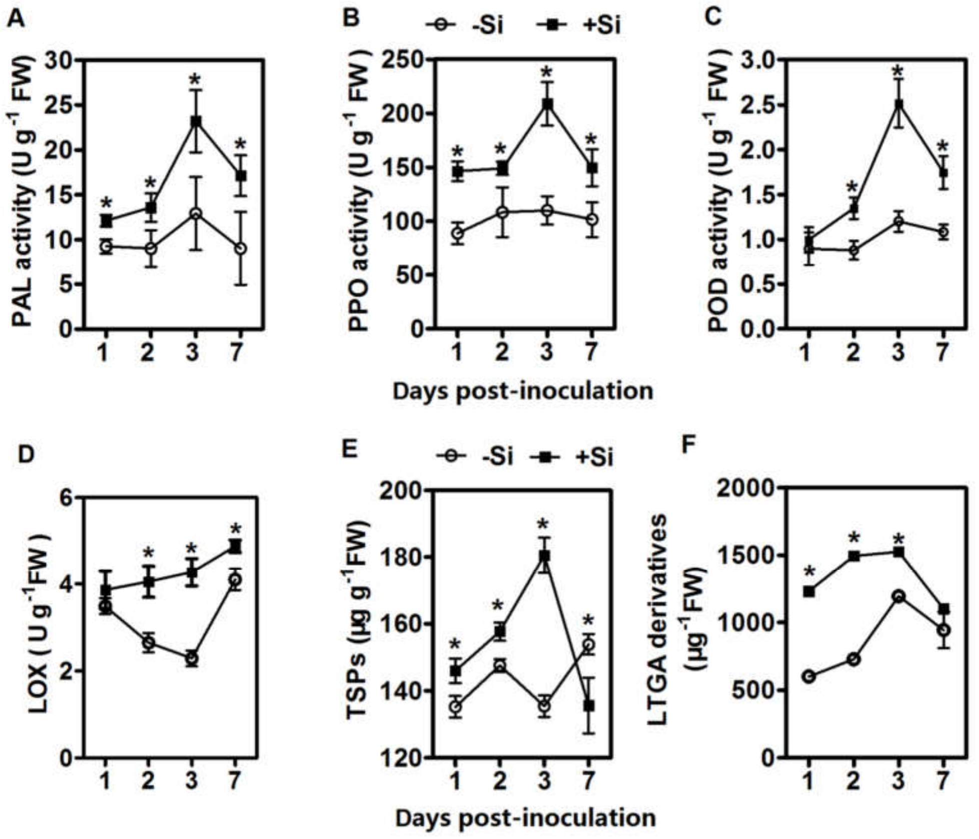

Figure 1. Effect of Si treatment on resistance related enzymes, total soluble phenols (TSPs) and lignin-thioglycolic acid (LTGA) derivatives in tomato root. Activities of PAL **(A)**, POD **(B)**, PPO **(C)**, and LOX **(D)** in root tissue of Si-treated (+Si) and non-treated (-Si) tomato plants at different times after inoculation with *R. solanacearum*. Concentrations of TSPs **(E)** and LTGA derivatives **(F)** in tomato root were detected after pathogen infection. Data are means ± SE of three replicate samples. Means followed by an asterisk are significantly different from untreated control at the same time-point (Student's *t*-test, $p < 0.05$). FW = fresh weight.

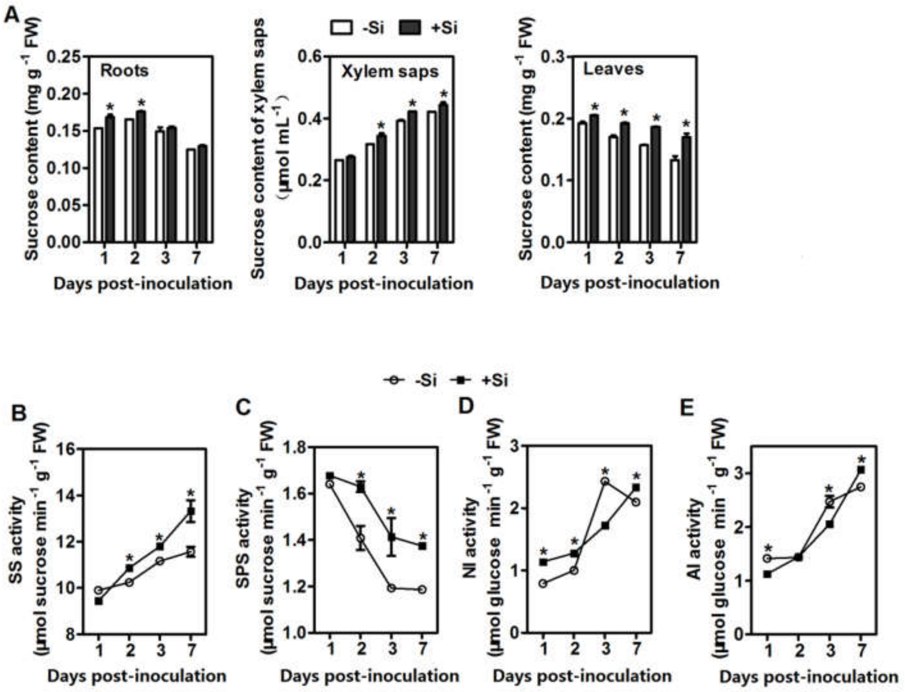

Figure 2. Effect of Si treatment on sucrose concentration and activities of enzymes related to sucrose metabolism in tomato at different time points after infection. **(A)** sucrose contents in roots, xylem saps and leaves of Si-treated (Si+) and untreated (Si-) tomato plants. The SS **(B)**, SPS **(C)**, NI **(D)** and AI **(E)** activities in leaf extracts in the same samples. AI, acid invertase; NI, neutral invertase; SS, sucrose synthase; SPS, sucrose-phosphate synthase. Values are the mean ± SE of three replicate samples. Means followed by an asterisk are significantly different from untreated control at the same time-point (Student's *t*-test, $p < 0.05$). FW = fresh weight.

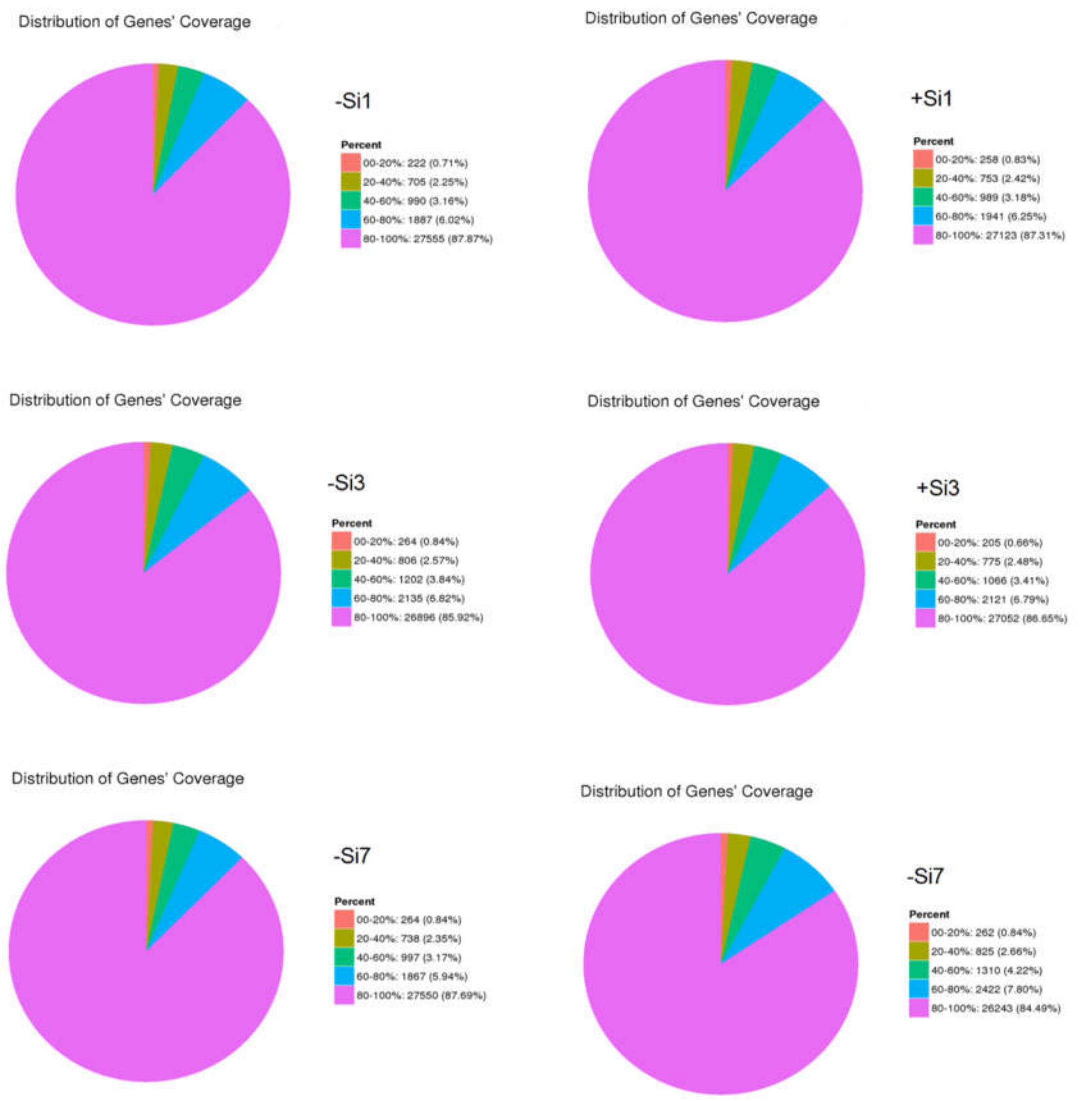

Figure 3. The distribution of gene coverage in six library. +Si1, +Si3 and +Si7 represents Si-treated samples obtained at 1, 3 and 7dpi, respectively; -Si1, -Si3 and -Si7 represents untreated samples obtained at 1, 3 and 7dpi, respectively.

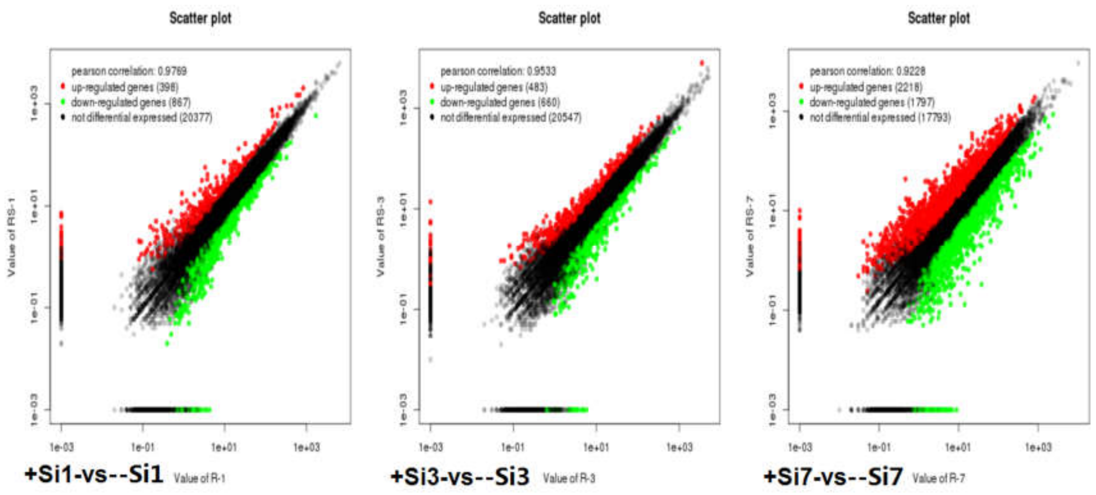

Figure 4. Scatter plot of differentially expressed genes in each comparison. +Si1, +Si3 and +Si7 represents Si-treated samples obtained at 1, 3 and 7dpi, respectively; -Si1, -Si3 and -Si7 represents non-Si-treated samples obtained at 1, 3 and 7dpi, respectively.

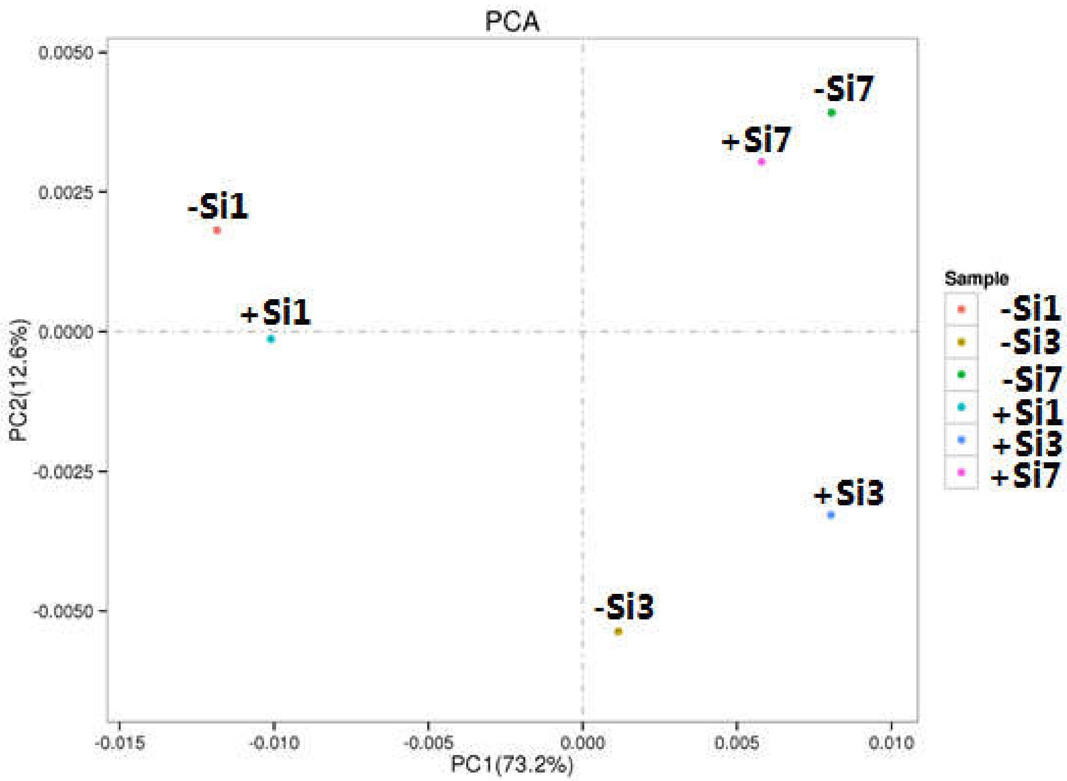

Figure 5. Principal component analysis (PCA) for all RNA-Seq samples. +Si1, +Si3 and +Si7 represents Si-treated samples obtained at 1, 3 and 7dpi, respectively; -Si1, -Si3 and -Si7 represents non-Si-treated samples obtained at 1, 3 and 7dpi, respectively.

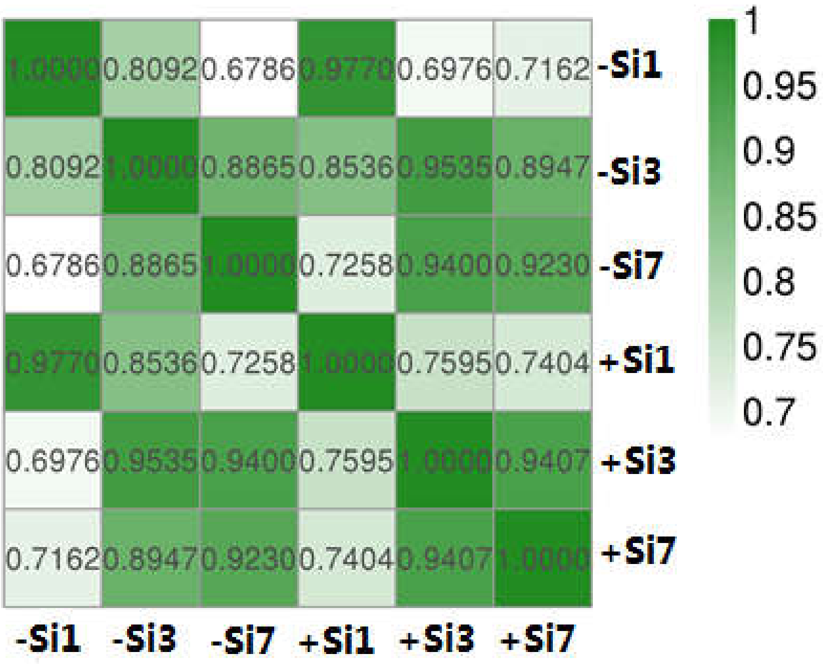

Figure 6. The Pearson correlation coefficients between six samples. +Si1, +Si3 and +Si7 represents Si-treated samples obtained at 1, 3 and 7 dpi, respectively; -Si1, -Si3 and -Si7 represents non-Si-treated samples obtained at 1, 3 and 7 dpi, respectively.

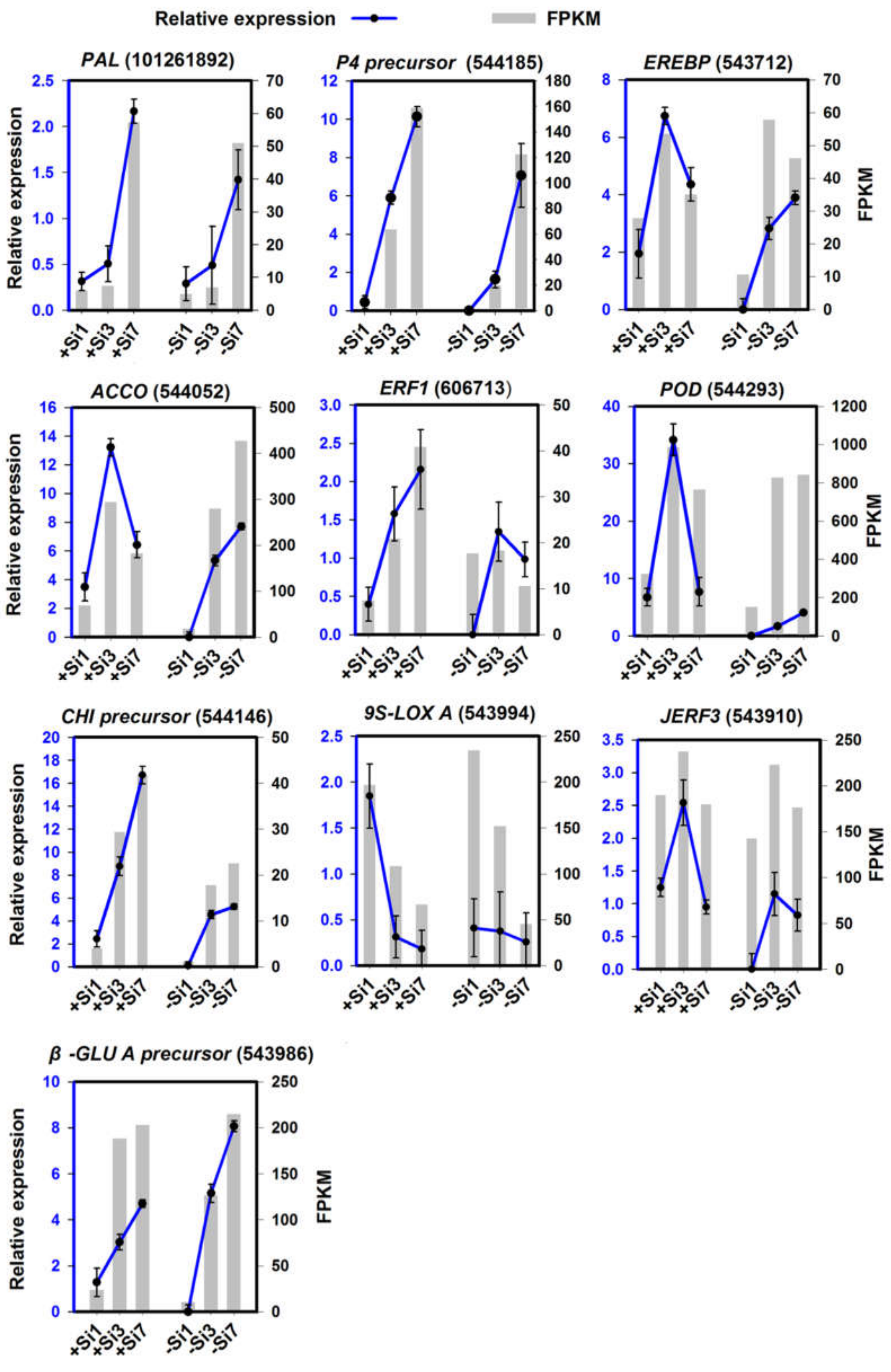

Figure 7. Expression of the selected 10 genes revealed by RNA-Seq and qRT-PCR. The expression levels obtained by qRT-PCR are represented in blue lines, qRT-PCR data showed the mean values from three replicates and the error bars represent the SE of the means, while the corresponding expression data for RNA-seq are represented in gray histogram. +Si1, +Si3 and +Si7 represents Si-treated samples obtained at 1, 3 and 7dpi, respectively; -Si1, -Si3 and -Si7 represents non-Si-treated samples obtained at 1, 3 and 7dpi, respectively; *PAL*: *phenylalanine ammonia-lyase*; *P4 precursor*: *pathogenesis-related leaf protein 4 precursor*; *EREBP*: *ethylene responsive element binding protein*; *ACCO*: *1-Aminocyclopropane-1-carboxylic acid oxidase*; *ERF1*: *ethylene response factor 1*; *POD*: *peroxidase*; *CHI precursor*: *chitinase precursor*; *9S-LOX*: *linoleate 9S-lipoxygenase A*; *JERF3*: *Jasmonate and ethylene responsive factor 1*; *β-GLU A precursor*: *glucan endo-1,3-beta-glucosidase A precursor*.

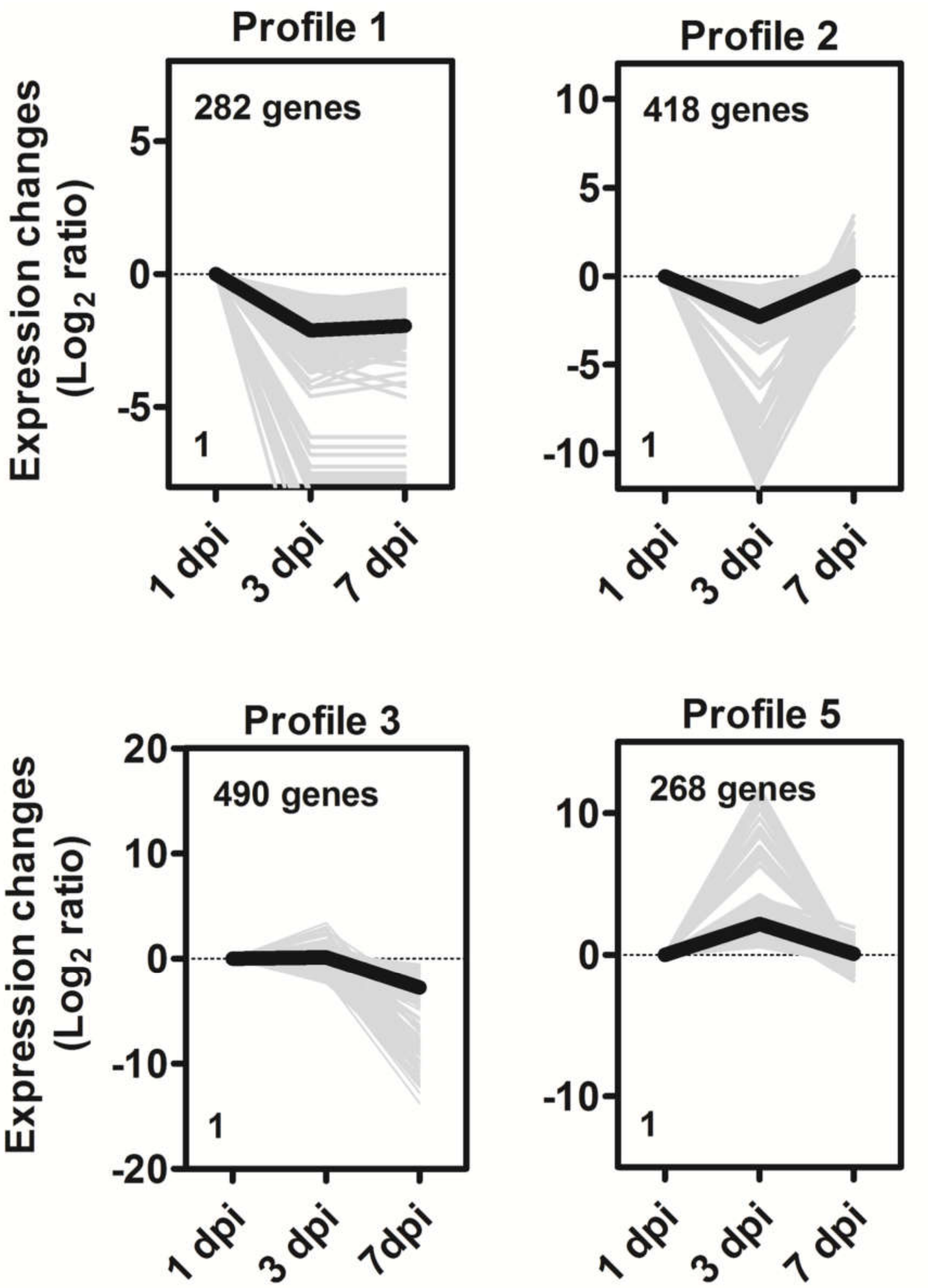

Figure 8. Expression profiles of the four statistically non-significant clusters (profile 1, 2, 3 and 5). X- and Y-axes represent days after *R. solanacearum* inoculation (dpi) and log2 fold change in gene expression between Si-treated and non-Si-treated samples, respectively. The top left hand corner indicates the number of DEGs belonging to each profile. The lower left hand corner contains the *p*-value of each profile.
